# Interactions between neutrophil extracellular traps and macrophages: the key to inflammatory diseases

**DOI:** 10.3389/fimmu.2026.1731687

**Published:** 2026-03-06

**Authors:** Xiaoyu Shan, Xiaodong Fan, Xiaofei Geng, Yongchun Liang, Yingxi Yang, Junping Zhang

**Affiliations:** 1 First Teaching Hospital of Tianjin University of Traditional Chinese Medicine, Tianjin, China; 2 National Clinical Research Center for Chinese Medicine, Tianjin, China; 3 College of Traditional Chinese Medicine, Tianjin University of Traditional Chinese Medicine, Tianjin, China; 4 Information and Technology School, Tianjin College of Commerce, Tianjin, China; 5 Tianjin Key Laboratory of Modern Chinese Medicine Theory of Innovation and Application, Tianjin, China

**Keywords:** cellular communication, immune inflammation, innate immunity, macrophages, neutrophil extracellular traps

## Abstract

Interactions between neutrophil extracellular traps (NETs) and macrophages play a critical role in the initiation and progression of inflammatory diseases. NETs regulate macrophage polarization and function by releasing components such as DNA, histones, and granule proteins, as well as by activating multiple signaling pathways. In turn, macrophages modulate the formation and clearance of NETs through the secretion of cytokines and proteases. This bidirectional interaction forms a positive feedback loop in autoimmune diseases, cardiovascular diseases, and the tumor microenvironment, exacerbating inflammatory responses and tissue injury. Investigating the specific mechanisms underlying the NETs–macrophage interplay may provide novel targeted therapeutic strategies for inflammatory diseases. Therefore, this article systematically reviews the mechanisms of NETs–macrophage interactions and their pathological roles in various inflammatory diseases, aiming to offer a theoretical foundation and translational potential for future research.

## 1 Introduction

Inflammation serves as a common pathological basis for the onset and progression of various diseases. Chronic inflammation not only induces tissue damage but also promotes tumor proliferation and metastasis (1, 2). In this process, neutrophils and macrophages, as crucial effector cells of the innate immune system, synergistically regulate the initiation, amplification, and resolution of inflammatory responses through intricate interactions (3, 4). Neutrophils, as the primary responders to inflammation, are rapidly recruited to the lesion site following tissue damage (5). Upon arrival, neutrophils not only phagocytose pathogens and cellular debris but also eliminate microorganisms by releasing granule contents with bactericidal effects, such as lysozyme and antimicrobial peptides (6). Additionally, neutrophils can release extracellular traps (NETs) to capture and kill pathogens (7).NETs are web-like structures primarily composed of decondensed chromatin and granular proteins. Under physiological conditions, these structures facilitate the capture and elimination of pathogens; however, excessive NET release represents a potential factor contributing to inflammation and tissue injury. Indeed, numerous studies have demonstrated that NETs play a central role in the pathophysiology of various inflammatory diseases, rendering them a critical therapeutic target in the management of inflammatory conditions (7). Macrophages represent another pivotal player in the inflammatory microenvironment, and their functional plasticity profoundly influences disease progression (8). Traditionally, the functional states of macrophages have been simplified into two polarization paradigms: M1 (pro-inflammatory) and M2 (anti-inflammatory/reparative), providing an initial framework for understanding their roles in immune responses (9). However, recent advances in single-cell technologies and multi-omics studies have revealed that macrophage activation is not a binary process but rather a continuum encompassing multiple intermediate phenotypes with dynamic spatiotemporal variations (10). This high degree of heterogeneity and plasticity is finely regulated by the tissue microenvironment, stimulatory signals, metabolic status, and epigenetic reprogramming (11). In inflammatory diseases, the functional states of macrophages are often closely associated with the formation and release of NETs, constituting the “NETs-macrophage axis,” which jointly orchestrates the initiation, amplification, and resolution of inflammation.

**Figure 1:**
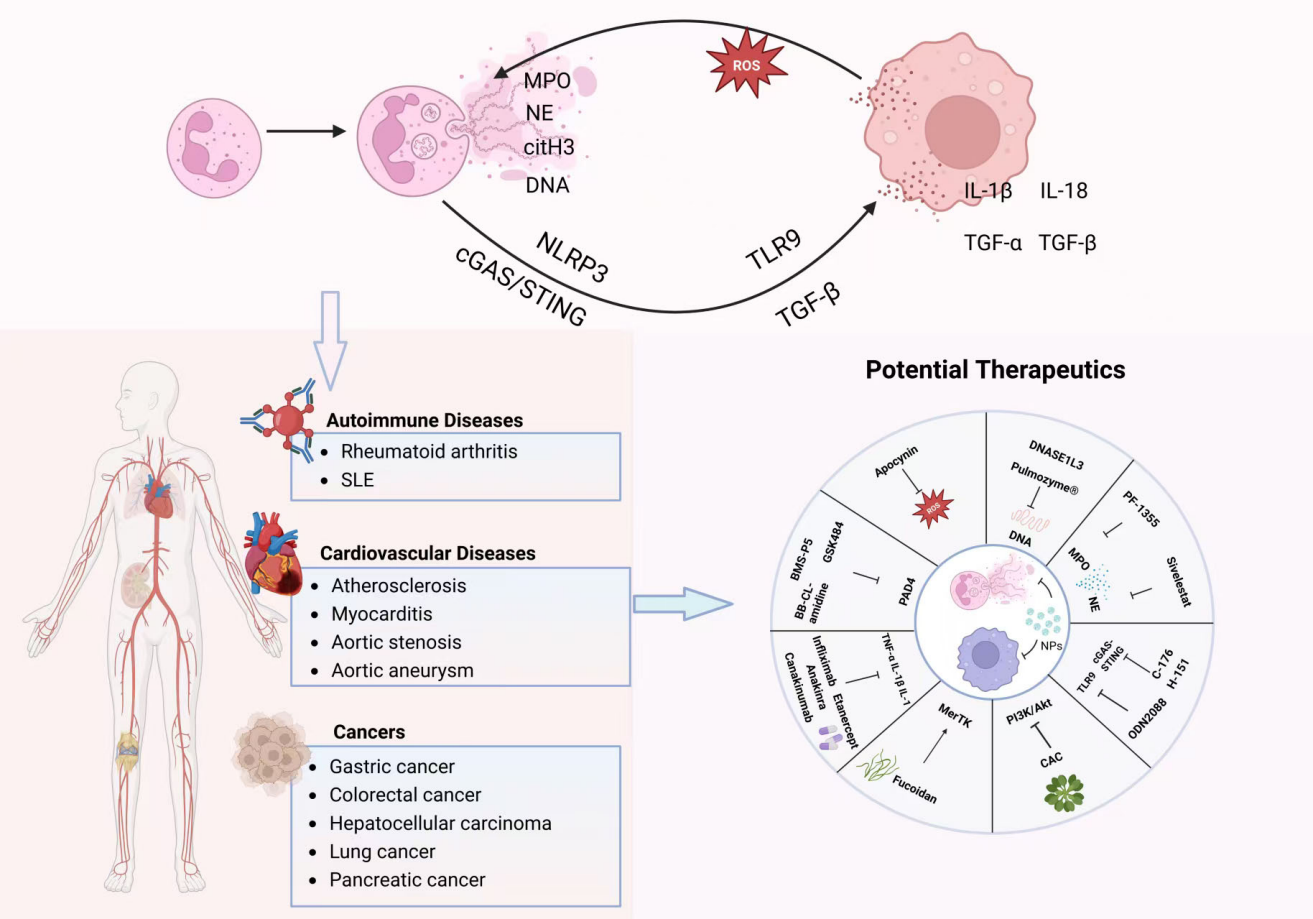
The NETs-macrophage axis: mechanisms in disease and targeted therapeutic strategies. NETs are released by neutrophils and primarily consist of DNA, myeloperoxidase (MPO), neutrophil elastase (NE), and citrullinated histone H3 (citH3). They can polarize macrophages via signaling pathways such as cGAS–STING, NLRP3, TLR9, TGF−β, and reactive oxygen species (ROS), promoting the secretion of factors including IL−1β, IL−18, and TGF−α/β, thereby establishing a pro−inflammatory positive−feedback loop. This NETs−macrophage axis is extensively involved in the pathogenesis of various diseases, including autoimmune disorders (e.g., rheumatoid arthritis, systemic lupus erythematosus), cardiovascular conditions (e.g., atherosclerosis, myocarditis, aortic stenosis, aortic aneurysm), and cancers (e.g., gastric cancer, colorectal cancer, hepatocellular carcinoma, lung cancer, pancreatic cancer). Therapeutic strategies targeting this axis may be developed from multiple perspectives, such as inhibiting NETs formation, promoting NETs clearance, regulating macrophage phenotypes, and employing combined delivery approaches.

In recent years, accumulating evidence has demonstrated the critical role of the NETs-macrophage axis in various inflammatory diseases. In autoimmune diseases, excessively produced NETs expose their internal autoantigens (such as DNA and histones) to the immune system (12). Macrophages phagocytose these NETs and present the autoantigens to T cells, which subsequently activate B cells to produce large quantities of autoantibodies (13, 14). These autoantibodies, in turn, bind to autoantigens, forming immune complexes that further stimulate additional NET formation and polarize macrophages toward a pro-inflammatory phenotype, leading to sustained amplification of inflammation and tissue damage (15–17). In cardiovascular diseases such as atherosclerosis, NETs formed within the vascular wall act as damage-associated molecular patterns (DAMPs), strongly activating macrophages and promoting their transition to a pro-inflammatory phenotype. This exacerbates local inflammation, foam cell formation, and plaque instability, ultimately increasing the risk of thrombosis (18–20). Within the tumor microenvironment, the interaction between NETs and tumor-associated macrophages (TAMs) exhibits a “double-edged sword” effect. NETs can recruit and modulate the functional state of macrophages, inducing characteristics that promote tumor progression, facilitate tumor immune evasion, and enhance angiogenesis and tumor metastasis (21, 22). In other contexts, however, they may also activate anti-tumor immunity (23, 24). Here, we summarize the latest evidence elucidating the interactions between NETs and macrophages, with a focus on their involvement in the pathogenesis of autoimmune diseases, cardiovascular diseases, and cancer. We further untangle the central mechanisms underlying immune dysregulation, sterile inflammation, and tumor immunity associated with these interactions. Additionally, potential therapeutic strategies targeting NETs or macrophages to mitigate the onset and progression of these diseases are discussed (**Figure 1**).

## 2 Formation and clearance mechanisms of NETs in inflammation

**Figure 2:**
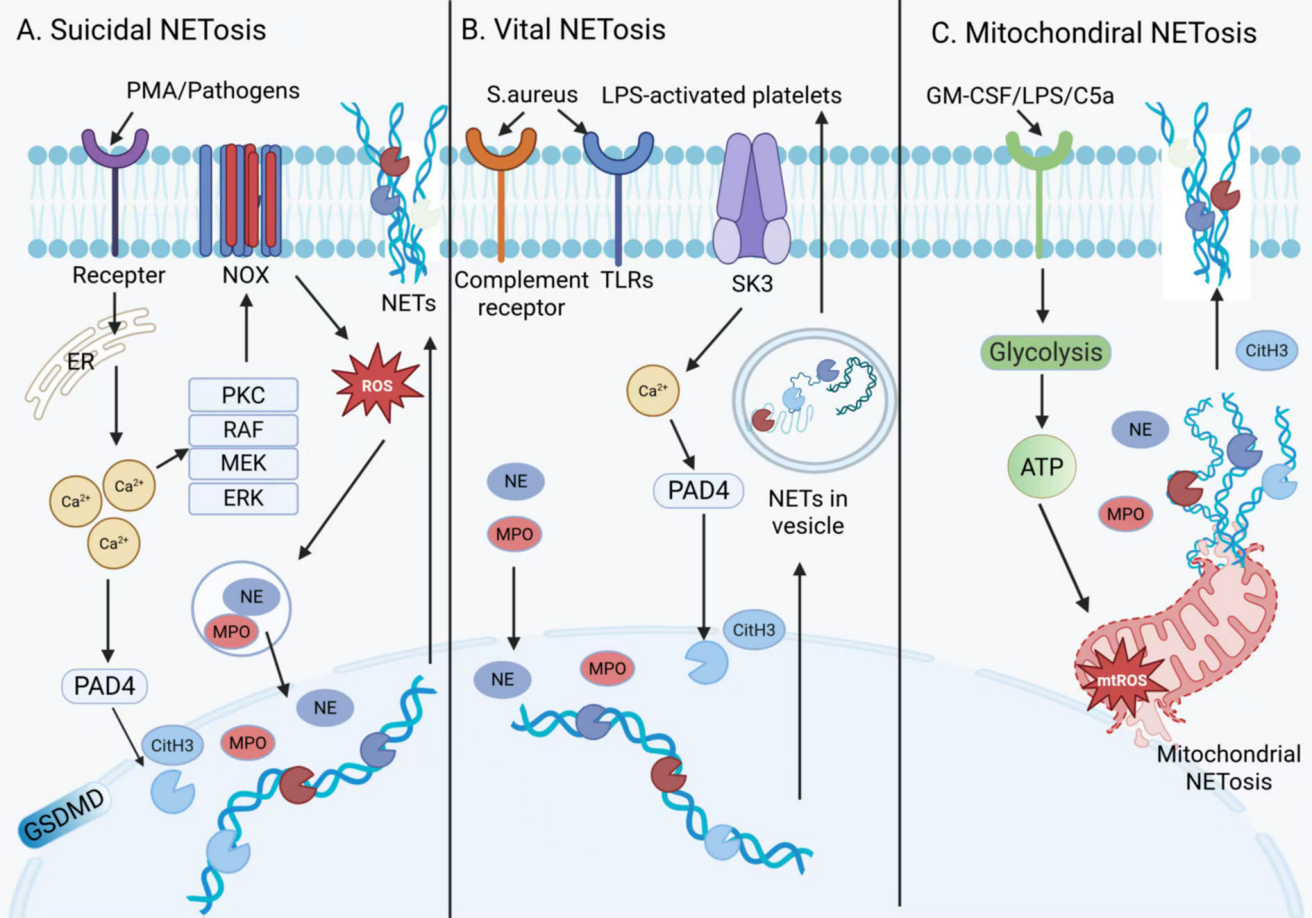
Mechanisms of NETs formation. NETs formation mechanisms. NETs formation can be primarily categorized into three types. The first pathway is **(A)** suicidal NETosis. PMA and pathogens (such as bacteria) activate cell surface receptors, triggering a signal cascade that activates NADPH oxidase (NOX), producing reactive oxygen species (ROS). ROS activate NE and MPO, while PAD4 mediates histone citrullination, leading to chromatin disassembly. Concurrently, GSDMD cleavage forms plasma membrane pores, causing nuclear membrane rupture and the release of NETs, accompanied by cell death. Another mechanism is referred to as **(B)** vital NETosis, which does not depend on NOX and ROS. Staphylococcus aureus or lipopolysaccharide (LPS)-activated platelets activate PAD4, which mediates histone H3 citrullination. After chromatin disassembly, it binds to granule proteins and releases NETs via vesicle encapsulation, while neutrophils remain viable and continue their immune functions. The final mechanism is termed **(C)** Mitochondrial NETosis. Granulocyte-macrophage colony-stimulating factor (GM-CSF) combined with LPS or C5a stimulation, dependent on glycolytic ATP and ROS, leads to mitochondrial DNA binding with granule proteins to form NETs, with no cell rupture occurring during this process.

The process by which neutrophils release NETs is termed NETosis. Unlike apoptosis and necrosis, NETosis in neutrophils represents a distinct form of programmed cell death triggered by various stimuli. The triggering factors are diverse, including phorbol myristate acetate (PMA), cytokines, autoantibodies, metabolic products such as cholesterol crystals and platelet, various microbial components including bacteria, fungi, parasites, and viruses, as well as physical and chemical stresses such as hypoxia and reactive oxygen burst, and abnormal immune signaling (25). Different stimuli activate distinct upstream signaling pathways, ultimately leading to the release of NETs in various forms. At present, based on cellular outcomes and the source of DNA, NETosis can be broadly classified into three types: suicidal NETosis, vital NETosis, and mitochondrial NETosis, each regulated by distinct molecular pathways (**Figure 2**).

The classical pathway of NETosis is the NADPH oxidase (NOX)-dependent mechanism, commonly referred to as “suicidal NETosis.” This pathway is typically accompanied by neutrophil rupture and cell death. This process is triggered by stimuli such as PMA (26)and microbial pathogens (27), which activate cell surface receptors. Subsequently, calcium ions released from the endoplasmic reticulum bind to and activate protein kinase C (PKC). PKC, in conjunction with the RAF-MEK-ERK-MAPK signaling cascade, promotes the assembly and activation of the NOX complex, leading to a burst of reactive oxygen species (ROS) (28). Elevated ROS levels trigger the activation and translocation of neutrophil elastase (NE) and myeloperoxidase (MPO), ultimately resulting in plasma membrane rupture and the extracellular release of NETs (25). Meanwhile, Peptidyl arginine deiminase 4(PAD4) is activated in the presence of calcium ions and converts arginine residues on histones (such as H3 and H4) into citrulline, thereby neutralizing the positive charges of histones and disrupting their electrostatic interactions with DNA. This process drives extensive chromatin decondensation, which represents one of the key steps in NETs release (29–31). In addition, NE cleaves gasdermin D (GSDMD) to generate pore-forming fragments that compromise the integrity of both the nuclear and plasma membranes, allowing citrullinated histones and DNA to be expelled into the cytoplasm (32). These components are subsequently modified by granule-derived proteins and cytoplasmic enzymes to form mature NETs. Secondly, the alternative pathway for NET formation is independent of NOX and ROS initiation but relies on PAD4 activation, and is termed “vital NETosis.” Unlike suicidal NETosis, vital NETosis does not result in neutrophil death; neutrophils remain viable and retain some functionality after releasing NETs (33). Upon stimulation by Staphylococcus aureus or platelets activated by lipopolysaccharide(LPS), Ca^2+^ influx occurs, which activates PAD4 and induces chromatin decondensation (32, 34). After chromatin decondensation, the released DNA is modified by granular proteins and histones, and is subsequently enveloped by vesicles originating from the nucleus. When these vesicles are extruded, extracellular NETs are formed, while neutrophil viability is maintained (35). Finally, in addition to releasing nuclear DNA-rich NETs, neutrophils can also release substantial amounts of mitochondrial DNA(mtDNA), a process termed “mitochondrial NETosis.” Yousefi’s study demonstrated that GM-CSF/LPS-induced mitochondrial NETosis is dependent on ROS, whereas PAD4 is not essential, and the release of mtDNA occurs independently of the citrullination of nuclear histones (36). Mitochondrial NETosis differs markedly from suicidal NETosis in that it does not induce neutrophil lysis. Instead, it relies on ATP generated through glycolysis to regulate cytoskeletal remodeling, thereby promoting the release of mtDNA and granular proteins (37). MtDNA functions not only as a structural component of NETs but also as DAMPs recognized by cytosolic cyclic GMP-AMP synthase (cGAS). Through the stimulator of interferon genes (STING), it activates downstream inflammatory pathways such as type I interferons (IFNs), the NF-κB pathway or the NLRP3 inflammasome, thereby contributing to various inflammatory diseases including systemic lupus erythematosus (SLE), tumors, and hepatic ischemia-reperfusion injury (38–40). Notably, activation of the cGAS/STING pathway can in turn promote NETosis in a feedback manner, forming a self-amplifying inflammatory loop. These findings reveal the dual structural and signaling functions of mitochondrial DNA in the formation of NETs, as well as its critical role in proinflammatory pathological processes. In addition to the three major modes of NET formation described above, recent studies have shown that NETosis can also be triggered through other programmed cell death pathways. Under stimulation by viruses or endotoxins, NETosis may occur via the necroptotic pathway mediated by receptor-interacting protein kinase (RIPK) and mixed lineage kinase domain-like protein (MLKL). Similar to mtNETosis, this process is independent of PAD4 and may result in cell death, representing a distinct form of suicidal NETosis (41). This further underscores the mechanistic diversity of NETosis. Understanding this diversity is essential for developing precise strategies to intervene in aberrant NETosis across different diseases.

The formation of NETs is primarily regulated by the aforementioned several mechanisms, and their clearance is also strictly controlled at multiple levels, involving complex molecular mechanisms and intercellular interactions, including degradation by extracellular Deoxyribonuclease (DNase) and phagocytosis mediated by various cells. DNase, including DNase I and II, which are produced and secreted by various cells (such as pancreatic cells), serve as the first line of defense in clearing NETs (42). DNases can specifically degrade the DNA within NETs, thereby disrupting their structure, impairing their ability to trap pathogens, and subsequently promoting the decomposition and clearance of NETs (43, 44). Macrophages, in turn, can more effectively recognize and phagocytose these fragmented NETs through surface receptors, thereby preventing their excessive accumulation and subsequent tissue damage (42). Reduced DNase activity or impaired macrophage function leads to defective NETosis clearance, resulting in abnormal accumulation of NETs within tissues. This accumulation exacerbates inflammatory responses and disease progression, exhibiting disease-specific pathogenic patterns across different conditions. For example, the buildup of NETs provides self-antigens that intensify autoimmune responses, promotes rupture of atherosclerotic plaques and thrombus formation, and creates a microenvironment conducive to tumor metastasis (20, 45, 46).

In summary, the formation and clearance of NETs represent a complex process involving multiple molecular mechanisms and intercellular interactions. The coordinated operation of these mechanisms is crucial for maintaining tissue homeostasis and preventing excessive inflammatory responses and tissue damage. A deeper understanding of the mechanisms underlying NET formation and clearance not only contributes to elucidating the pathophysiology of various diseases, particularly inflammatory diseases, but also provides a theoretical foundation for the development of targeted therapeutic strategies.

## 3 Interactions between NETs and macrophages

**Figure 3:**
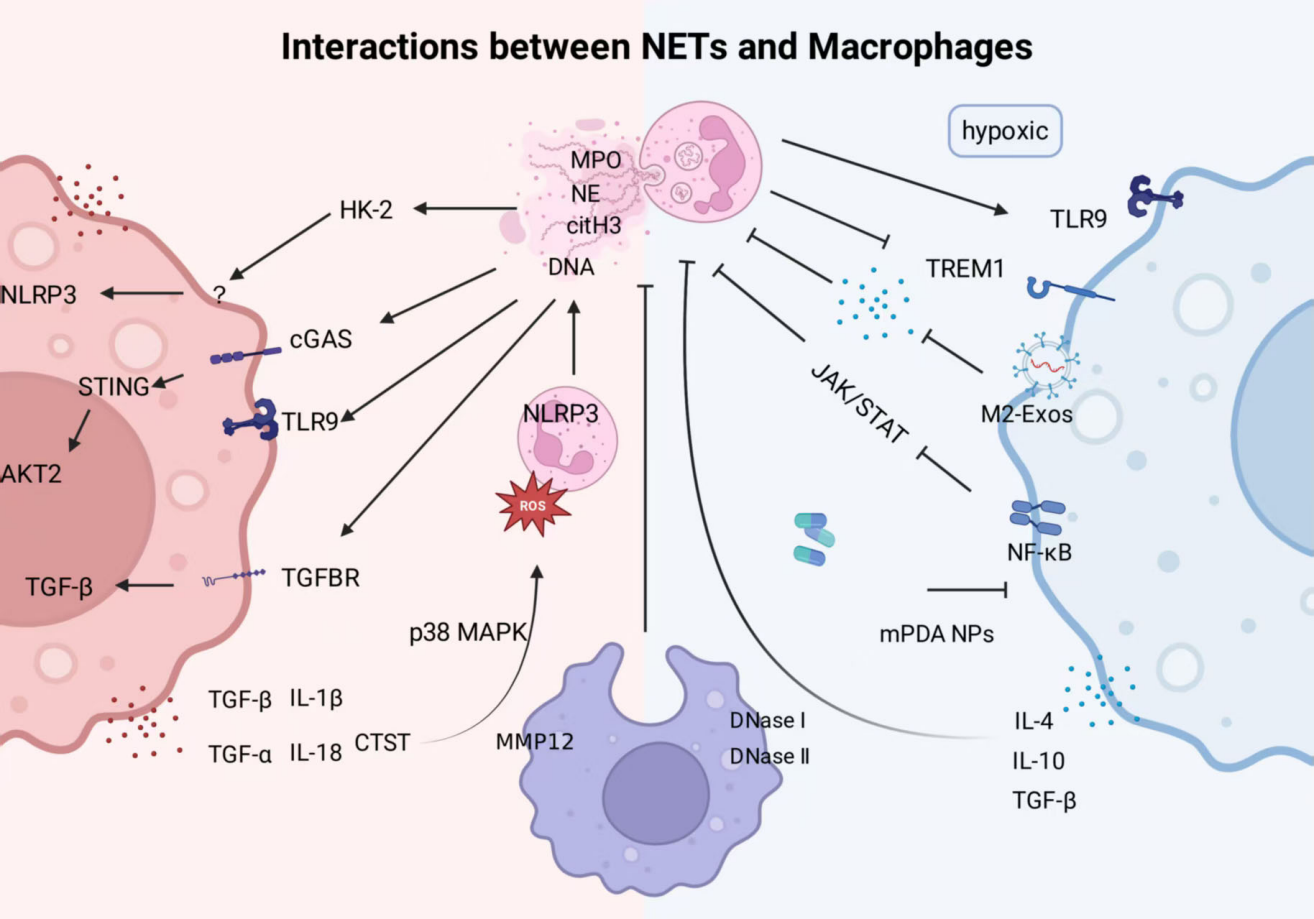
Interactions between NETs and macrophages. This figure depicts the context-dependent, bidirectional crosstalk between NETs and macrophages and the feedback circuits it establishes. Left panel (red): Under acute infection or inflammatory flare conditions, NET–macrophage interactions form a proinflammatory positive-feedback loop. NETs polarize macrophages toward a proinflammatory phenotype via HK-2 upregulation, cGAS–STING signaling, TLR9 activation, and modulation of TGF-β pathways. In turn, proinflammatory macrophages amplify NETosis by releasing IL-1β, TNF-α, and cathepsin C, thereby escalating inflammation. Right panel (blue): In specific microenvironments (e.g. hypoxia or the tumor milieu), NET–macrophage interactions shift toward anti-inflammatory or pro-repair programs. Under hypoxia, NETs can drive macrophage polarization to an antiinflammatory phenotype through TLR9; TREM1 signaling also contributes. Antiinflammatory phenotype macrophages secrete IL-4, IL-10, and TGF-β and release M2-derived exosomes (M2-Exos) that suppress neutrophil NETosis. The schematic further indicates that mesoporous polydopamine nanoparticles (mPDA NPs), used as macrophage-mimetic nanotherapies, curb NET formation by inhibiting NF-κB and JAK/STAT signaling, providing a negative-feedback brake. Center: Macrophage clearance and degradation of NETs. Macrophages recognize and engulf NETs and mediate enzymatic digestion via DNase I/DNase II (DNA backbone) and MMP-12 (protein components), a process essential for tissue homeostasis and for preventing persistent inflammation.

Interactions among immune cells constitute a complex and finely tuned regulatory network that not only determines the intensity and direction of immune responses but also profoundly influences tissue homeostasis and disease progression (33). Among these interactions, the crosstalk between NETs and macrophages represents one of the core mechanisms underlying inflammation and immune regulation (33). Under physiological conditions, NETs and macrophages act in coordination: upon activation, neutrophils release NETs, which in turn stimulate macrophages to polarize toward a phenotype with enhanced clearance capacity, thereby facilitating the timely removal of NETs, effective pathogen elimination, and subsequent tissue repair (47, 48). However, under pathological conditions, impaired NETs clearance or their persistent formation can lead to a vicious cycle with macrophages. NETs promote macrophage polarization toward a proinflammatory phenotype, while dysfunctional macrophages fail to efficiently clear NETs, resulting in amplified inflammatory signaling, hindered tissue repair, and even adverse outcomes such as fibrosis and tumor metastasis. Together, these processes drive the progression of diseases including rheumatoid arthritis (RA), atherosclerosis, and cancer (49–51).This section aims to integrate existing studies and establish a unified conceptual model to elucidate how the two components mutually regulate each other through specific molecules and pathways under different pathophysiological conditions, thereby forming positive or negative feedback loops that ultimately determine the direction of the immune response. The following discussion will focus on three aspects: the contextual regulation of macrophage function by NETs, the active regulation of NETs formation by macrophages, and the macrophage-mediated clearance and degradation of NETs (**Figure 3**).

### 3.1 Contextual regulation of macrophage function by NETs

The functional regulatory relationship between NETs and macrophages has been a recent focus in immunological research. Substantial evidence indicates that NETs significantly influence macrophage polarization and phagocytic function through their unique structures and components; however, such regulation is highly context-dependent.

During the peak of inflammation caused by acute infection or sterile injury, NETs primarily drive macrophages to polarize into a pro-inflammatory phenotype resembling the classical M1 type, thereby amplifying the inflammatory response to facilitate clearance of threats. This phenomenon has been observed in various diseases and pathological models in existing studies. In studies of gouty arthritis, *in vitro* experiments revealed that MSU crystal–induced NETs markedly promoted macrophage polarization toward the M1 phenotype, as evidenced by increased expression of CD86, CD80, iNOS, and NLRP3 inflammasome–related proteins. Mechanistic investigations indicated that NETs might drive this process through upregulation of HK-2, while silencing or inhibiting HK-2 reversed these effects, suggesting that HK-2 plays a pivotal role in this pathway. Validation in animal models demonstrated that blocking NETs formation with the PAD4 inhibitor Cl-amidine or degrading NETs with DNase I significantly alleviated joint swelling and reduced local infiltration of M1-type macrophages, further supporting the role of NETs in promoting macrophage M1 polarization during gouty inflammation. However, these interventions themselves may exert nonspecific effects, and the animal model cannot fully replicate the complex pathophysiological processes of human disease (52). In the osteoporosis model of ovariectomized (OVX) mice, NETosis of bone marrow neutrophils was found to be significantly activated. *In vitro* experiments further confirmed that NETs induced M1 polarization of RAW264.7 macrophages and promoted osteoclastogenesis. Mechanistically, NETs may exert their effects by activating the cGAS–STING/AKT2 signaling pathway: knockdown of STING inhibited NETs-induced osteoclast differentiation, whereas overexpression of AKT2 partially restored this process, suggesting that cGAS–STING functions upstream of AKT2. It should be noted that this mechanism has so far been demonstrated mainly in cell line experiments (such as RAW264.7), in which long-term passaging may lead to phenotypic drift. The generalizability of these findings to *in vivo* pathology, as well as their associations with age and sex, remains to be further validated (53). The Mertk ^-^ MHC-II ^lo/int^ macrophages subset has been shown in myocardial infarction models to differentiate toward a proinflammatory phenotype through the NETs–TLR9 signaling pathway. Oligodeoxynucleotide 2088 (ODN 2088) is a potent Toll-Like Receptor 9 (TLR9) inhibitor that specifically blocks TLR9 receptors on the surface of immune cells to suppress excessive inflammatory activation. The use of the TLR9 inhibitor ODN 2088 or TLR9-deficient macrophages effectively abolishes the NETs-induced polarization, demonstrating that TLR9 is an essential receptor in this process (54). Although *in vivo* experiments provide supporting evidence, the specific DNA sequences or modifications responsible for TLR9 pathway activation remain unclear, the definition and function of the specific Mertk^-^ MHC-II^lo/int^ subpopulation require further validation across diverse disease contexts. In a trauma/hemorrhagic shock (T/HS) model, *In vitro* experiments further showed that NETs promoted M1 polarization by suppressing the transforming growth factor-β (TGF-β) signaling pathway mediated by TGFBR2, contributing to T/HS-induced intestinal barrier dysfunction (55). Overall, during the inflammatory flare phase of various diseases such as gouty arthritis and myocardial infarction, most studies support that NETs induce pro-inflammatory macrophages and may establish a negative feedback loop that amplifies inflammation and accelerates disease progression. However, it should be noted that these conclusions are highly dependent on specific disease models and experimental conditions, and clinical data from patients remain relatively limited, making it difficult to determine whether these mechanisms are universally applicable to all forms of acute inflammation in humans.

However, in tissue repair and specific pathological microenvironments such as tumors or hypoxic conditions, NETs also demonstrate the ability to polarize toward states characterized by anti-inflammatory, reparative, or tumor-promoting features, analogous to the classical M2 phenotype or its subgroups. In experiments simulating myocardial ischemia *in vitro* under hypoxia, NETs promote macrophage differentiation towards the M2b subgroup, characterized by upregulated IL-10 expression, while blocking TLR9 signaling can reverse this effect (47). In light of this, it is inferred that the TLR9 pathway in the regulation of macrophage polarization by NETs may be microenvironment-related, activating macrophages to transform into different states under different conditions. However, the specific regulatory mechanism remains unclear, and the current simple *in vitro* hypoxia model is insufficient to simulate the complex cell interaction network *in vivo*, requiring further validation *in vivo* and in clinical settings. In systemic sclerosis (SSc) mice model, NETs promoted fibrotic progression by inducing M2-like macrophage differentiation. Inhibiting NET formation or depleting neutrophils reduced M2 macrophage numbers and alleviated disease severity (56). Notably, M2 macrophages themselves exhibit significant functional heterogeneity, encompassing diverse subgroups involved in processes such as tissue repair, fibrosis, or immunosuppression. NETs can also reprogram monocyte differentiation fate: neutrophil NE contained in NETs cleaves IL-4Rα, thereby inhibiting monocyte differentiation into dendritic cells and instead promoting their transformation into an anti-inflammatory macrophage phenotype (57). Current research often relies on a limited set of markers for their characterization, potentially oversimplifying their intricate phenotypic complexity. M2 polarized macrophages play a complicit role in the tumor microenvironment, promoting tumor progression through multiple pathways. In the stomach cancer (GC) model, NETs may be involved in promoting the polarization of tumor-associated macrophages (TAMs) toward an M2-like phenotype, thereby supporting tumor growth. This finding has been validated in both *in vivo* and *in vitro* experiments. In GC mice or cells with TREM1 knockout, tumor growth, neutrophil infiltration, NETs marker levels, and M2 macrophage marker levels were all suppressed. Degradation of NETs using DNase I reversed the TREM1 overexpression-induced increases in NETs formation and M2 macrophage expression. However, the association between NETs and M2 macrophages has not yet been extensively validated across tumor models of different types or stages. The high heterogeneity of the tumor microenvironment suggests that the role of NETs may vary substantially depending on cancer type, disease stage, and individual differences. Therefore, their generalizability and clinical relevance require further confirmation (58). At present, most studies on the interaction between NETs and macrophages are derived from *in vitro* co-culture experiments, and whether this interaction has a direct causal association with disease chronicity remains to be clarified with additional evidence. Nonetheless, under certain conditions, NETs can indeed drive macrophages toward an anti-inflammatory phenotype, warranting further investigation in clinical and animal disease models in the future.

The influence of NETs on macrophage phagocytic function is likewise context-dependent. During acute infection or in the early stages of tumor development, several studies have shown that NETs can enhance phagocytosis through physical trapping or by modulating signaling molecules. However, these phenomena are mostly observed under specific experimental conditions. For instance, in early hepatic metastasis models, NETs exert anti-metastatic effects by downregulating the “don’t eat me” signal CD24 on the surface of tumor cells. In contrast, in advanced-stage tumors, NETs have been reported to promote metastasis, underscoring the complexity of NETs’ functional roles (48, 59). On the other hand, in chronic inflammation, excessive NET release may impair macrophage phagocytosis, leading to the accumulation of NETs. m6A methylation has been shown to regulate sepsis by inhibiting both NET formation and macrophage phagocytosis, suggesting that aberrant NET accumulation can disrupt phagocytic function (60). Several studies have reported that dysregulated NET formation exacerbates inflammation and tissue injury, which may indirectly compromise macrophage phagocytic capacity. In acute lung injury (ALI), platelet-activated neutrophils form excessive NETs, which impair fundamental functions of alveolar macrophages by activating the coagulation cascade and sustaining the release of pro-inflammatory mediators (61).

In summary, the regulation of macrophage polarization and phagocytic function by NETs is not fixed; rather, it is highly dependent on the specific characteristics of the inflammatory stage and tissue microenvironment, exhibiting a dual role that ranges from cooperative defense to driving pathology.

### 3.2 Active regulation of NETs formation by macrophages

Macrophages do not passively respond to NET regulation; rather, they actively participate in NET formation. The polarization state, phagocytic function, and macrophage extracellular trap (MET) formation are key factors modulating NET generation.

NET formation is closely associated with macrophage polarization. Pro-inflammatory macrophages enhance NETosis through multiple mechanisms, including the secretion of pro-inflammatory cytokines and the regulation of oxidative stress, whereas anti-inflammatory or reparative macrophages inhibit the formation of NETs by secreting anti-inflammatory factors or suppressing oxidative stress. First, the secretion of pro-inflammatory cytokines is a major pathway through which pro-inflammatory macrophages promote NET generation. In *in vitro* studies of GA, it was observed that macrophages release large amounts of the key inflammatory cytokine Interleukin-1β (IL-1β) upon stimulation with MSU crystals. Interestingly, although IL-1β alone does not directly induce neutrophils to release NETs, it markedly amplifies NETs formation triggered by MSU crystals. Specifically, in the presence of IL-1β, MSU crystal–stimulated neutrophils release greater amounts of extracellular DNA, accompanied by a significant increase in the secretion of MPO, NE, and other NETs-specific complexes. This potentiating effect can be completely abolished by an IL-1β receptor antagonist, confirming that macrophage-derived IL-1β serves as a pivotal mediator regulating this process (62). However, in clinical practice, IL-1β inhibitors (such as anakinra) do not always produce consistent therapeutic efficacy in patients with gout, and a considerable proportion of individuals show poor or no response. This observation suggests that, beyond the macrophage-IL-1β-NETs axis, additional critical pathways are likely involved, and that *in vitro* models may oversimplify this complexity. In a mouse model of hepatitis induced by concanavalin A, macrophages activated by NETs were found to produce pro-inflammatory cytokines such as IFNs, which in turn enhance the NETosis capacity of neutrophils, forming an immune amplification loop (63). Similarly, other pro-inflammatory cytokines secreted by macrophages, including IL-6, IL-18, and TNF-α, can also induce NET formation (33). Second, the level of oxidative stress regulates NET generation. Studies have shown that S100A9-deficient neutrophils exhibit elevated mitochondrial reactive oxygen species (mtROS) levels in the macrophage microenvironment, leading to increased “suicidal NETosis.” This suggests that macrophages may indirectly promote NET formation by modulating the oxidative stress status of neutrophils (64). Alveolar macrophages serve as key initiators of NET formation during pulmonary ischemia–reperfusion injury. By secreting mediators such as cathepsin C (CTST), they activate neutrophils and the NLRP3 inflammasome, thereby driving NETosis. Histones released from NETs can, in turn, reactivate M1-type macrophages, forming a positive feedback loop that amplifies inflammation (65). In LPS-induced acute lung injury, this mechanism has been further elucidated: macrophage-derived CTSC activates proteinase 3 on the neutrophil membrane, triggering a burst of ROS production and extensive NET formation via the IL-1β/p38 MAPK axis. Activated neutrophils can also secrete CTSC in an autocrine manner, establishing a self-amplifying loop that exacerbates tissue injury (66). Although direct evidence from patient samples is still lacking, both *in vivo* and *in vitro* experiments indicate a bidirectional promotive interaction between macrophages and NETs, constituting a self-reinforcing inflammatory cycle that collectively drives the progression of lung injury. In summary, although evidence from patient is still lacking, both *in vivo* and *in vitro* experiments have demonstrated that macrophages promote the formation of NETs. This process is closely linked to the NET-induced polarization of macrophages toward a proinflammatory phenotype, forming a characteristic positive feedback loop that amplifies inflammation.

However, macrophages can also exhibit inhibition of NETosis. Exosomes derived from M2 macrophages (M2-Exos) exhibit anti-inflammatory activity in inflammatory diseases and inhibit excessive NET formation, thereby protecting organ function (67). This indicates that macrophage-mediated regulation of NETs is phenotype-dependent. Subsequent studies revealed that M2-Exos, enriched with anti-inflammatory factors such as TGF-β and IL-10, promote neutrophil apoptosis and inhibit the transition of endothelial cells to a pro-inflammatory phenotype, thereby blocking neutrophil recruitment and the triggering signals for NETosis, ultimately suppressing NET formation. Therapeutic strategies that employ cell membrane–mimicking nanotechnology to target and modulate the immune microenvironment have attracted considerable attention in the field of inflammatory diseases (68, 69). MM@mPDA-PM NPs represent an innovative biomimetic nanodrug delivery system in which mesoporous polydopamine nanoparticles (mPDA NPs) loaded with Peimine (PM) are encapsulated by macrophage membranes (MMs), thereby achieving active targeting of pulmonary inflammatory sites. This system not only effectively eliminates reactive oxygen species and alleviates oxidative stress but also modulates macrophage polarization toward the M2 phenotype, suppresses the NF-κB and JAK/STAT signaling pathways, and markedly reduces the expression of NETs-related markers such as MPO, NE, and PAD4, ultimately mitigating excessive inflammation and tissue injury in acute lung injury (70).This effect echoes the role of NETs in inducing M2 polarization under certain conditions, potentially forming a negative feedback or homeostatic loop that helps to limit excessive inflammation and promote tissue repair.

Beyond polarization-dependent regulation, macrophage phagocytic function and METs also indirectly influence NET formation. A primary function of macrophages is to phagocytose senescent or apoptotic cells, and uncleared cellular debris can activate neutrophils to form NETs. Impaired phagocytic function of macrophages leads to defective clearance of apoptotic cells, indirectly promoting NET release. In periodontitis, insufficient expression of histone deacetylase SIRT6 in macrophages results in failure of inflammation resolution and exacerbation of periodontitis, accompanied by accumulation of apoptotic neutrophils (ANs) and increased NET generation. Mechanistically, high glucose stimulation disrupts the SIRT6–miR-216/217 axis in macrophages, impairing their ability to clear apoptotic cells and indirectly promoting NET release (71). Additionally, macrophages themselves can release structures similar to NETs, known as METs, which often act synergistically with NETs in disease pathology (72, 73). In atherosclerosis, oth METs and NETs contribute to thrombosis and inflammation. In rheumatoid arthritis (RA), METs promote autoantibody production and autoimmune responses by forming stable autoantigen–DNA complexes, while NETs have been shown to drive the pathophysiology of the disease; together, they exacerbate pathological injury. In summary, macrophages are key regulators of NETs formation, with their effects—either promoting or inhibiting—dependent on their functional state and phenotype. Together with NETs, they constitute a dynamic and bidirectional immune regulatory network.

### 3.3 Clearance and degradation of NETs by macrophages

Macrophages serve not only as regulators of NET formation but also as the primary effectors responsible for NET clearance. By phagocytosing and degrading NETs, macrophages maintain immune homeostasis and prevent persistent inflammatory responses.

The phagocytosis of NETs by macrophages is not a passive process; it begins with precise “recognition.” A repertoire of pattern recognition receptors and scavenger receptors expressed on the macrophage surface specifically recognize histones on the NET scaffold and embedded proteins such as MPO and NE (65, 74). This recognition is often not silent; it frequently triggers macrophage activation, leading to the secretion of pro-inflammatory cytokines, indicating that NET clearance by macrophages is an integral part of immune modulation (75, 76). Following recognition, macrophages efficiently “phagocytose” and “degrade” NETs. Through endocytosis, NET fragments are engulfed to form phagosomes. Subsequently, cytoplasmic exonucleases, including DNases, degrade NET components, ensuring thorough clearance to prevent sustained immune activation (42, 77). In this process, DNase II plays a critical role by hydrolyzing the DNA backbone, while various proteases cooperatively degrade granular proteins.

In pro-inflammatory M1 macrophages, MMP-12 plays a central role in NET degradation and acts as a key mediator driving macrophage-mediated NET clearance (78). This multifunctional protease not only degrades complement C3 to prevent excessive complement system activation but also neutralizes the pro-inflammatory anaphylatoxins C3a and C5a, thereby mitigating inflammation. Moreover, MMP-12 enhances macrophage phagocytic efficiency by cleaving complement fragments iC3b and C3b and modulating complement activity to avoid dysregulated immune activation. This has been validated in a mouse model of abdominal aortic aneurysm. MMP-12-deficient mice exhibited elevated levels of complement component C5a and NETs, while administration of a complement inhibitor reduced NETosis and aortic rupture, suggesting that MMP-12 may influence NET clearance via regulation of the complement system (79). Additionally, the AMP-activated protein kinase (AMPK) pathway significantly enhances macrophage clearance of NETs, offering a potential therapeutic avenue for acute respiratory distress syndrome (ARDS) (80). *In vitro* experiments, Resolvin T4 (RvT4) was observed to markedly increase intracellular cAMP and phosphorylated AMPK levels in human macrophages. When protein kinase A (PKA) or AMPK inhibitors were applied, RvT4-stimulated NET uptake was completely blocked, indicating that RvT4 activates macrophage NET clearance function via the cAMP–PKA–AMPK signaling axis (81). This mechanism was further confirmed in mouse *in vivo* studies, demonstrating that RvT4 significantly enhances the capacity of macrophages to phagocytose and clear NETs by activating the AMPK signaling pathway, thereby exerting protective effects in inflammatory models such as sepsis.

Macrophages actively clear and degrade NETs through specific recognition and efficient phagocytosis mediated by key factors centered on MMP-12 and AMPK. This process is not only crucial for maintaining tissue homeostasis but also constitutes an integral part of fine immune regulation.

## 4 Mechanisms of NET-macrophage interactions in inflammatory diseases

### 4.1 NETs and macrophages in autoimmune diseases

#### 4.1.1 Rheumatoid arthritis: NETs-mediated heterogeneity and osteoclastic differentiation of macrophages

As a typical representative of autoimmune inflammatory diseases, RA provides an ideal model for elucidating the complex interactions between NETs and macrophages in pathological environments. In RA, NETs and macrophages form a multi−layered interaction network, jointly driving the pathological processes of joint inflammation, synovial hyperplasia, and bone destruction (49).

The post-translational modifications of NETs play a crucial role in the immunogenicity and pathological persistence of RA (82). Specifically, histone citrullination mediated by peptidylarginine deiminases (PAD2 and PAD4) creates the key autoantigen citrullinated histone H3 (citH3). Notably, citrullination is heterogeneous; the cellular distribution and substrate specificity of different PAD family members vary, leading to citrullination at distinct arginine residues on different proteins. This results in a diverse “citrullinome,” which significantly influences the specificity and affinity spectrum of autoantibodies (83, 84). In addition to citrullination, NETs undergo other modifications, such as carbamylation of LL37 (carLL37), which collectively broaden the spectrum of autoantibody recognition (85, 86). Importantly, these diverse modifications render NETs resistant to DNase digestion, allowing them to evade extracellular degradation by macrophages. This resistance prolongs their half-life, continuously providing antigenic stimulation and leading to subsequent necrosis, thereby exacerbating inflammation (87). Antigens such as citH3 released from NETs are presented via Human Leukocyte Antigen – D Related (HLA-DR), which drives the production of anti-citrullinated protein antibodies (ACPAs) (83). In turn, ACPA-immune complexes feedback-activate neutrophils and macrophages, establishing a positive inflammatory feedback loop. Regarding bone destruction, citH3 directly promotes osteoclast differentiation through the TLR4-MyD88 signaling pathway (88, 89). Clinical data indicate a positive correlation between synovial citH3 levels and bone resorption (85). Moreover, subsequent animal experiments demonstrated that the bone-eroding effect of carbamylated NETs (cNETs) is strictly dependent on TLR4 (85).

The response of macrophages to NETs is both dynamic and multifaceted. Research has shown that Rab5a plays a crucial role in mediating the endocytosis of NETs by macrophages. The NE contained within NETs activates the Rab5a-NF-κB signaling pathway, subsequently promoting the secretion of inflammatory cytokines by macrophages (90). In a chronic inflammatory environment, these pro-inflammatory cytokines synergize with receptor activator of nuclear factor kappa-B ligand (RANKL) to facilitate the differentiation of M1-type macrophages into osteoclasts (89). Single-cell sequencing has identified a specific population of CD14+CD68+TRAP+ cells in the synovium of RA patients, which concurrently express high levels of osteoclast markers and M1-related factors, indicating a direct cellular source of bone erosion (91). At the same time, the capacity of macrophages to clear modified NETs is significantly impaired. Tolerance to DNase results in the accumulation of NETs and subsequent necrosis, which releases additional DAMPs that further exacerbate inflammation (87, 92, 93).

In summary, the interaction between NETs and macrophages in RA exhibits distinct autoimmune disease specificity. NETs acquire immunogenicity and DNase resistance through the heterogeneity of citrullination and multiple modifications, leading to impaired clearance and persistence. These NETs drive the autoimmune cycle and, by activating the pro-inflammatory osteoclastogenic transformation of macrophages, collectively promote RA-associated inflammation and bone destruction, constituting a profound pathological core and therapeutic target.

#### 4.1.2 Systemic lupus erythematosus: impaired NETs clearance and systemic immune amplification loop

SLE is characterized by systemic inflammation and the presence of disease-specific anti-double-stranded DNA (anti-dsDNA) autoantibodies (94). The interaction between NETs and macrophages plays a pivotal role in amplifying systemic inflammation and impairing the clearance of autoantibodies. This pathological cycle begins with the generation of NETs that possess unique pathogenic properties in SLE. NETs derived from SLE patients are enriched not only in citrullinated autoantigens and anti-dsDNA antibodies but also contain aberrant components such as endogenous retroviral (ERV) proteins, collectively forming a highly immunogenic autoantigen reservoir (13, 95). However, factors such as reduced serum DNase I activity, the presence of DNase inhibitors, and anti-NET antibodies in SLE hinder degradation of the DNA backbone of NETs (96). Additionally, NETs themselves may directly suppress macrophage efferocytosis, further aggravating defective clearance (97). These persistently retained NETs continuously activate macrophages through multiple mechanisms. Nucleic acids and protein components carried by NETs engage pattern recognition receptors such as Toll-like receptors (TLRs) to initiate inflammatory signaling (13, 98). Forkhead box O3 (FOXO3) is a key downstream target of the PI3K/AKT signaling pathway, playing a central role in regulating processes such as the cell cycle, apoptosis, oxidative stress responses, and autophagy (99). Studies found that elevated levels of miR-122-5p in SLE patient-derived exosomes inhibit the homeostasis regulator FOXO3, thus releasing the restraint on the NF-κB pathway and synergistically promoting macrophage polarization toward a pro-inflammatory M1 phenotype (100). NET-associated proteins, such as LL-37, further drive excessive activation of the NLRP3 inflammasome in neighboring macrophages, resulting in massive inflammatory cytokine release (101). Activated M1 macrophages, in turn, secrete large amounts of inflammatory mediators and present NET-derived autoantigens, thereby inducing plasmacytoid dendritic cells (pDCs) to produce high levels of type I interferons, especially IFN-α. IFN-α facilitates dendritic cell maturation, T cell activation, and B cell autoantibody production, leading to the formation of immune complexes (102–104). These complexes deposit in organs such as the kidneys, skin, and vasculature, triggering complement activation and type III hypersensitivity reactions, which culminate in characteristic multi-organ damage. Furthermore, the inflammatory milieu, particularly enriched in IL-18 and IFN-α, stimulates neutrophils to produce more NETs and impairs endothelial repair, reinforcing the pathological cycle (105). This forms a self-perpetuating loop—persistent NET accumulation, sustained macrophage activation, amplified autoimmune responses, progressive tissue injury, and further NET production—that underpins the chronic and systemic nature of SLE.

**Figure 4:**
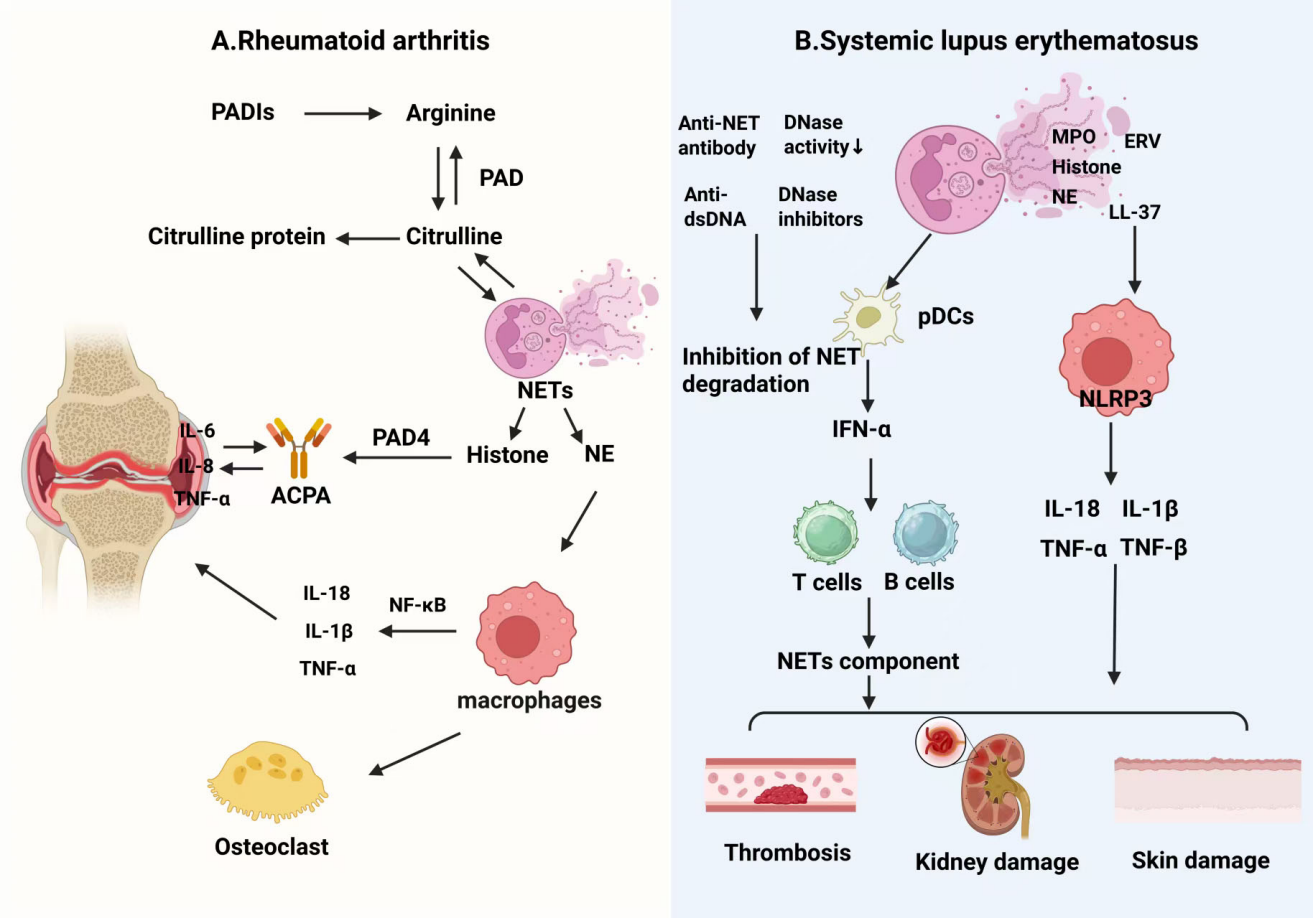
NETs and macrophages in autoimmune diseases. **(A)** In rheumatoid arthritis (RA), local post-translational modifications and bone erosion are key features. In the joint, PAD-mediated citrullination promotes NET formation. These modified NETs act as autoantigens, activate macrophages via NF-κB to release TNF-α, and directly promote osteoclast differentiation, driving joint damage. **(B)** In systemic lupus erythematosus (SLE), defective NET clearance and a type I interferon storm are central. Due to reduced DNase activity and anti-NET antibodies, NET components (MPO, LL-37, histones) accumulate. They activate pDCs to produce IFN-α and stimulate macrophages to release IL-18, IL-1β, and TNF-α, driving T/B cell responses and autoantibody production. Persistent NETs chronically activate type I IFN pathways, while NET components and anti-dsDNA antibodies deposit in organs, causing kidney and skin damage. NLRP3 inflammasome activation further amplifies inflammation. Both diseases share a vicious cycle: persistent NETs → macrophage activation → amplified autoimmunity → tissue injury → further NET formation.

In summary, the pathophysiological mechanisms in RA and SLE clearly demonstrate the central role and disease-specific characteristics of NET–macrophage interactions in autoimmune disorders (**Figure 4**). While NETs are originally designed to capture and kill pathogens, their excessive formation or impaired clearance in various autoimmune diseases releases large quantities of modified autoantigens and persistently activates immune cells, including macrophages, thereby driving and amplifying autoimmune responses (85, 106). Significant molecular insights have been gained, identifying critical roles for the PAD4, TLR4, and NLRP3 signaling pathways as well as defects in NET clearance. However, translating these basic research findings into effective therapeutic strategies that can break this cycle remains a formidable challenge. The future of targeted therapy will depend on accurately addressing the high cellular heterogeneity and achieving selective modulation of intercellular interactions between defined immune cell subsets.

### 4.2 NETs and macrophages in cardiovascular diseases

#### 4.2.1 Atherosclerosis: malignant inflammatory loop and plaque instability

Atherosclerosis (AS) is a chronic, lipid-driven inflammatory disease characterized by the formation, progression, and eventual rupture of atherosclerotic plaques. NETs released by activated neutrophils, together with macrophages, play pivotal pathophysiological roles at every stage of this process—from plaque initiation to destabilization and rupture (107–109).

Hypercholesterolemia and related stimuli activate the vascular endothelium, promoting the recruitment of neutrophils and platelets. Neutrophil–platelet aggregates then form, and platelet-derived mediators such as platelet-activating factor (PAF) and thromboxane A_2_ (TXA_2_) potently activate neutrophils, triggering the initial burst of NET release (110–112). Cholesterol crystals deposited within the vessel wall act as direct activators of the NLRP3 inflammasome, further inducing NETosis (113). The activated NLRP3 inflammasome, mediated by Caspase-1, promotes the cleavage and secretion of pro-IL-1β while driving GSDMD-mediated pore formation, ultimately triggering a distinct form of NETosis closely associated with the NLRP3-GSDMD pathway. This process is independent of the initial stimulation by neutrophil-platelet complexes, representing another crucial mechanism for the sustained generation of NETs within the plaque microenvironment. NET structural components disrupt the endothelial barrier, markedly increasing vascular permeability and facilitating low-density lipoprotein (LDL) infiltration and subsequent oxidation into oxLDL. OxLDL not only serves as a key ligand for macrophage scavenger receptors but also stimulates endothelial cells to secrete chemokines such as monocyte chemoattractant protein-1 (MCP-1), thereby recruiting monocytes/macrophages into the lesion (114, 115). Single-cell transcriptomic studies have revealed pronounced heterogeneity among plaque macrophages, including pro-inflammatory CCR2^+^ monocyte-derived macrophages, Trem2^+^ lipid-associated macrophages with reparative potential, and self-renewing tissue-resident subsets (116). Furthermore, NETs not only drive macrophages to shift toward a phenotype expressing pro-inflammatory genes (e.g., IL-1β, TNF-α), but also significantly upregulate the expression of their scavenger receptors (e.g., CD36) and enhance their phagocytic capacity. After engulfing large amounts of lipids, these macrophages transform into foam cells—a hallmark event in AS (117, 118). Activated foam cells themselves secrete substantial amounts of inflammatory cytokines such as IL-1β. These cytokines can recruit and activate more neutrophils to release NETs. Moreover, the intracellular accumulation of cholesterol (especially when the ABCA1/G1 efflux pathways are impaired) can directly activate the NLRP3 inflammasome in macrophages. Consequently, a self-sustaining positive feedback loop is formed: NETs - macrophage/foam cell activation - release of inflammatory factors/crystals - further NETosis (18, 109, 113). MerTK, a critical receptor for directing macrophage differentiation toward the M2c subtype and promoting the resolution of inflammation, becomes compromised in advanced plaques. NETs can trigger MerTK shedding via the HMGB1–TLR4–ADAM17 signaling axis, impairing efferocytosis, hindering apoptotic cell clearance, and promoting necrotic core expansion—all of which contribute to plaque instability and rupture (20, 119). Emerging evidence further suggests that metabolites within the plaque microenvironment (e.g., succinate, lactate), along with histone lactylation modifications, may fine-tune the dynamics of macrophage–NET interactions (116). In AS, NETs and macrophages establish a self-sustaining vicious cycle—initiated by lipid infiltration, perpetuated through foam cell formation and inflammatory amplification, and exacerbated by impaired repair processes—which collectively drive plaque onset, progression, and eventual rupture.

#### 4.2.2 Myocardial infarction, myocarditis, and valvular heart disease: common inflammatory synergistic destruction

Myocardial infarction (MI) elicits a robust sterile inflammatory response, driven in part by the reciprocal, deleterious crosstalk between NETs and macrophages during thrombus formation and ischemia-reperfusion injury (120). In murine MI models, plaque rupture exposes cholesterol crystals and collagen that further activate neutrophils, while abundant neutrophil–platelet aggregates formed during thrombosis potentiate excessive NET release (121). As potent mediators of inflammation and thrombosis, NETs skew macrophages toward pro-inflammatory phenotypes (e.g. early CCR2+ macrophages) and induce the production of cytokines such as IL-1β and TNF-α, thereby amplifying the inflammatory cascade (122). Simultaneously, NETs interfere with the clearance function of macrophages, leading to the accumulation of dead cell debris, hindering the resolution of inflammation and tissue healing, and increasing the risk of heart failure (123).

In viral myocarditis, NETs and M1 macrophages are also positively correlated, forming an amplified inflammatory circuit. In a murine model of viral myocarditis, the peak of NET formation coincides with the activation of pro-inflammatory macrophages. Genetic knockout experiments confirmed that blocking NET formation significantly reduced the secretion of IL-1β by cardiac macrophages, indicating that NETs are a key trigger for macrophage polarization (124). Furthermore, single-cell studies suggested the potential existence of a macrophage subpopulation expressing interferon-stimulated genes (ISGs) in myocarditis, although its interaction with NETs requires further elucidation (125).

In aortic valve stenosis, NETs activate macrophages and promote their polarization toward the M1 phenotype, inducing the release of pro-inflammatory cytokines (e.g., IL-1β) that exacerbate local inflammation and valvular fibrosis. Inhibition of NLRP3 activity also reduced the M1/M2 macrophage ratio, suggesting that NLRP3 activation is closely associated with M1 macrophage polarization (126). Therefore, NETs may indirectly regulate macrophage phenotype via NLRP3 activation.

**Figure 5:**
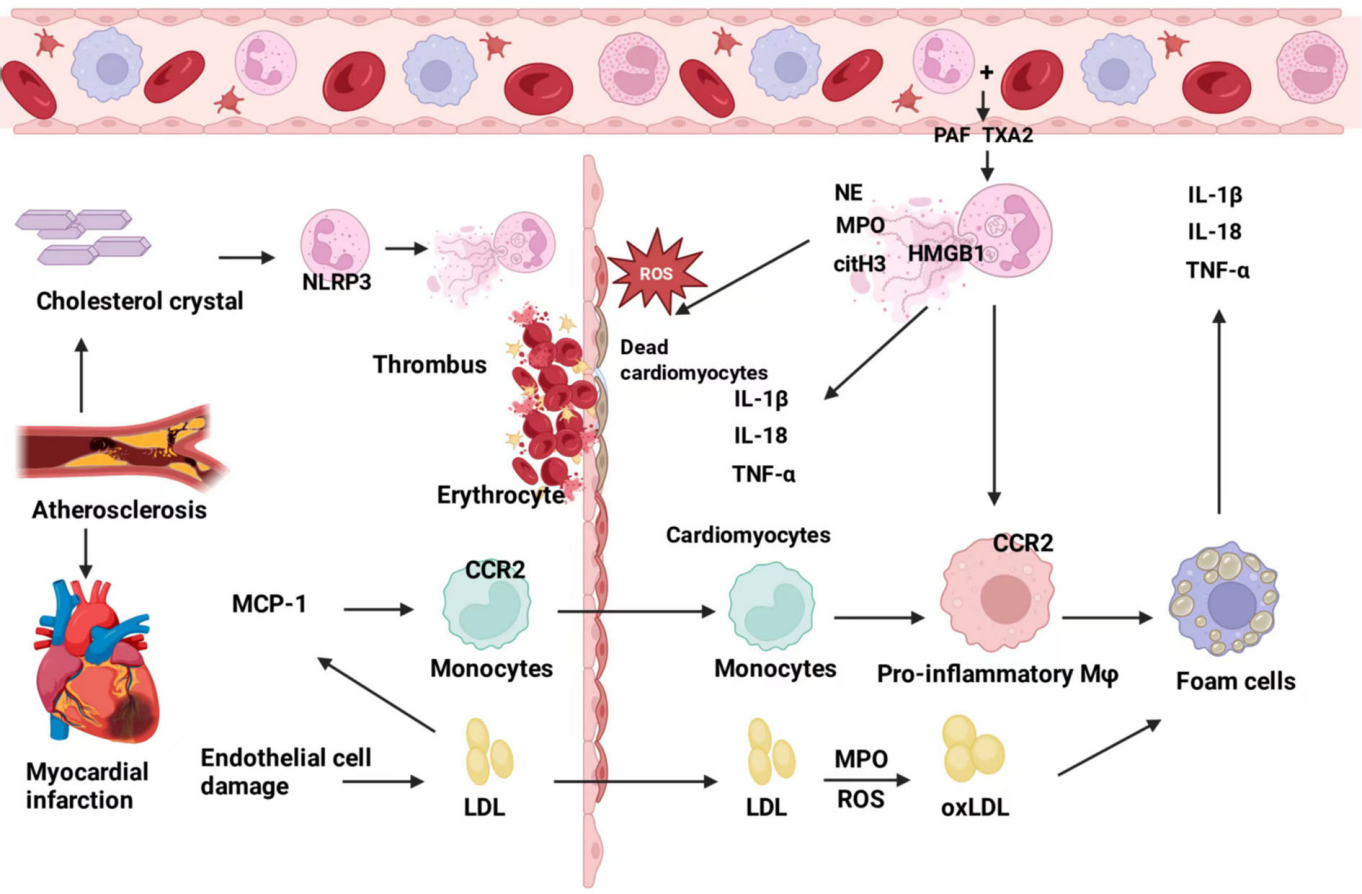
NETs and macrophages in atherosclerosis. Atherosclerosis (AS): Lipid infiltration and plaque instability are defining features. Upon endothelial activation, neutrophils and platelets form aggregates; platelet−derived PAF and TXA2 trigger early NET release, while cholesterol crystals directly activate the NLRP3 inflammasome to induce NETosis. NETs disrupt the endothelial barrier and increase permeability, facilitating LDL entry and MPO/ROS−dependent oxidation to ox−LDL. MCP−1 recruits monocytes, and NETs skew macrophages toward proinflammatory phenotypes that engulf lipids and become foam cells. Activated macrophages secrete IL−1β, TNF−α, and IL−18, further recruiting and activating neutrophils to amplify NET formation. In advanced plaques, NETs drive MerTK shedding via the HMGB1–TLR4–ADAM17 axis, impairing efferocytosis and resolution, expanding the necrotic core, and promoting plaque instability. Myocardial infarction (MI), myocarditis, and valvular disease: These entities share a thrombus/ischemia–reperfusion–driven cooperative injury paradigm. Plaque rupture and thrombosis robustly induce NETs, polarizing macrophages toward proinflammatory subsets (e.g., early CCR2^+^ macrophages) and elevating IL−1β and TNF−α, thereby intensifying local inflammatory “storms.” NETs also hinder macrophage clearance of dead cardiomyocyte debris, delaying resolution and repair and increasing heart−failure risk. In viral myocarditis, NET peaks coincide with M1 macrophage activation, and NET blockade reduces cardiac IL−1β. In aortic valve stenosis, NETs promote M1 polarization via NLRP3, driving inflammation and fibrosis.

NETs and macrophages constitute a mutually reinforcing vicious cycle in cardiovascular pathologies. NETs not only initiate inflammation and cause tissue damage but also drive macrophages toward a pro-inflammatory, highly phagocytic phenotype through epigenetic and metabolic reprogramming. In turn, activated macrophages further stimulate the formation of additional NETs. This self-amplifying pathological dialogue jointly promotes key processes ranging from atherosclerotic plaque destabilization to impaired repair after myocardial injury (**Figure 5**). Future studies should further integrate single−cell multi−omics, spatial transcriptomics, and metabolomics data to untangle the dynamic network of NET−macrophage interactions across different disease stages and microenvironments, thereby providing a basis for developing spatiotemporally specific immunomodulatory strategies.

### 4.3 NETs and macrophages in tumors

#### 4.3.1 The pro-tumor vicious cycle of NETs and macrophages

The tumor immune microenvironment (TIME) plays a crucial role in cancer initiation, progression, metastasis, and invasion, and is primarily composed of various immune cells, tumor cells, cytokines, and chemoattractants (107). NETs, generated by activated neutrophils, and macrophages are key components of the TME. Their impact on tumor progression is highly dependent on the specific pathological and therapeutic context.

NETs are pivotal in sculpting the immunosuppressive tumor microenvironment. Emerging evidence reveals that TAMs deviate from the classical M1/M2 paradigm, instead occupying a highly plastic continuum of intermediate states regulated by complex local signaling networks. NET-associated components—including DNA, histones, and granular proteins—function as DAMPs that reprogram infiltrating monocytes, directing their differentiation toward immunosuppressive and pro-angiogenic phenotypes. Through this mechanism, NETs shift the overall macrophage spectrum toward immunosuppression, thereby facilitating tumor progression and immune evasion (127, 128). This NET–TAM feedback loop has been consistently observed across diverse cancer types.

Components of NETs can act as potent signaling molecules that directly regulate macrophage function. Single-cell transcriptomics has delineated TAM subpopulations with distinct molecular programs—for example, SPP1+ TAMs with a senescence-associated secretory phenotype (SASP) signature, and ID1-high TAMs that sustain cancer stemness and restrict CD8+ T cell infiltration. NET-induced or NET-modulated TAMs typically exhibit immunosuppressive features, including high PD-L1 and arginase-1 expression, along with secretion of VEGF and TGF-β (129). Thus, NETs do not merely trigger an M2-like polarization; they remodel the macrophage state continuum toward immunosuppressive, pro-angiogenic, tumor-promoting phenotypes, fostering a microenvironment that supports tumor progression and immune evasion. When evaluating the prognostic and TME-predictive value of NETs-related risk scores in CRC patients, researchers observed that M0 and M2 macrophage infiltration was most prominent in high-risk CRC patients, and M2 macrophage levels positively correlated with NETs risk scores (130). Therefore, it is hypothesized that NETs are associated with M2 macrophage polarization within the tumor microenvironment of CRC (129). Src kinase-associated phosphoprotein 1 (SKAP1), an immune cell adaptor, has been identified as a novel colon cancer-associated gene. *In vitro* experiments on colon cancer, SKAP1-overexpressing colon cancer cells were found to effectively induce NET formation and secrete IL-4. Interleukin-4 (IL-4) is a prototypical anti-inflammatory cytokine known to polarize macrophages toward the M2 phenotype. Based on this, it has been hypothesized that NETs may similarly activate macrophages, driving their polarization toward an M2 phenotype and thereby promoting immunosuppression and tumor progression. In the aforementioned ex vivo GC experiments, we likewise observed that NETs drive macrophage polarization from an anti-tumor M1 state toward a pro-tumor M2 state, with TREM1 signaling acting upstream to promote NET formation (58). However, these observations are confined to ex vivo GC models and have not been broadly replicated in other tumor contexts or validated clinically. They therefore remain provisional under experimental conditions, and their generalizability and translational relevance require further confirmation.

In addition to NETs regulating macrophage function, macrophages can also mediate NET formation via the NLRP3 pathway or through the secretion of related pro-inflammatory factors, forming a vicious cycle that promotes cancer progression. Spleen tyrosine kinase (SYK) is an important target in liver-associated signaling pathways. Studies have shown that macrophages directly or indirectly promote neutrophil recruitment and NET formation through the SYK–NLRP3–IL-1β axis and secretion of the chemoattractants CXCL1/CXCL2, thereby exacerbating liver ischemia-reperfusion injury and tumor recurrence (131). Targeting SYK (e.g., using GS-9973) can simultaneously inhibit SYK activity in both macrophages and neutrophils, disrupting this “vicious cycle”and demonstrating therapeutic potential. *In vitro* experiments on lung cancer, when macrophages were co-cultured with A549 lung cancer cells and NETs were added, the metastatic and invasive capabilities of A549 cells were enhanced, which may be associated with pro-inflammatory cytokines (IL-6, IL-1β, IL-18, and TNF-α) released by macrophages (132). In the absence of macrophages, NETs were unable to directly promote A549 cell migration and invasion, indicating that the tumor-promoting effect of NETs depends on the presence of macrophages. Macrophages are the principal intermediaries through which NETs exert pro−invasive and pro−migratory effects on tumor cells. Accordingly, disrupting NETs, macrophages, or their crosstalk has emerged as a promising therapeutic strategy in lung cancer. Overall, evidence from most tumor models indicates that NET–macrophage interactions form a mutually reinforcing feed−forward loop that entrenches an immunosuppressive, tumor−promoting microenvironment and drives cancer progression.

#### 4.3.2 Anti-tumor potential of NETs

Although the prevailing view supports an association between the presence of NETs and tumor progression, a minority of studies have presented contrary findings. Under certain circumstances, NETs may also exert tumor-suppressive effects (133). For instance, in a colorectal cancer model, NETs induced by the combination of 5-fluorouracil (5-FU) and a glutaminase inhibitor were found to promote tumor cell apoptosis via the RAGE pathway through the release of cathepsin G, thereby inhibiting tumor growth (134). Ex vivo experiments conducted in head and neck squamous cell carcinoma and melanoma models have similarly demonstrated that NETs can exert anti-tumor effects by inducing apoptosis (135, 136). In a recent clinical study on lung cancer, it was found that NETs derived from both healthy individuals and lung cancer patients exhibited similar tumor cell-killing capabilities ex vivo. However, NETs from lung cancer patients were deficient in inhibiting tumor migration and even occasionally exhibited pro-migratory tendencies (137). These findings indicate that the function of NETs possesses significant duality and context-dependency, and their interactive network with macrophages is far more complex than a simple vicious cycle.

In summary, there exists a close and complex crosstalk between NETs and macrophages within the tumor immune microenvironment. Disrupting this vicious cycle between NETs and macrophages—whether by degrading NETs, inhibiting key signaling molecules, or reprogramming macrophages—offers promising directions for intervening in the tumor microenvironment and developing novel anti-cancer strategies. Notably, it should be acknowledged that, because NETs’ functions are context-dependent, any targeted intervention strategy must be precisely evaluated and optimized within the specific pathological and therapeutic context.

## 5 Potential therapeutic targets for NET-macrophage interactions

**Figure 6:**
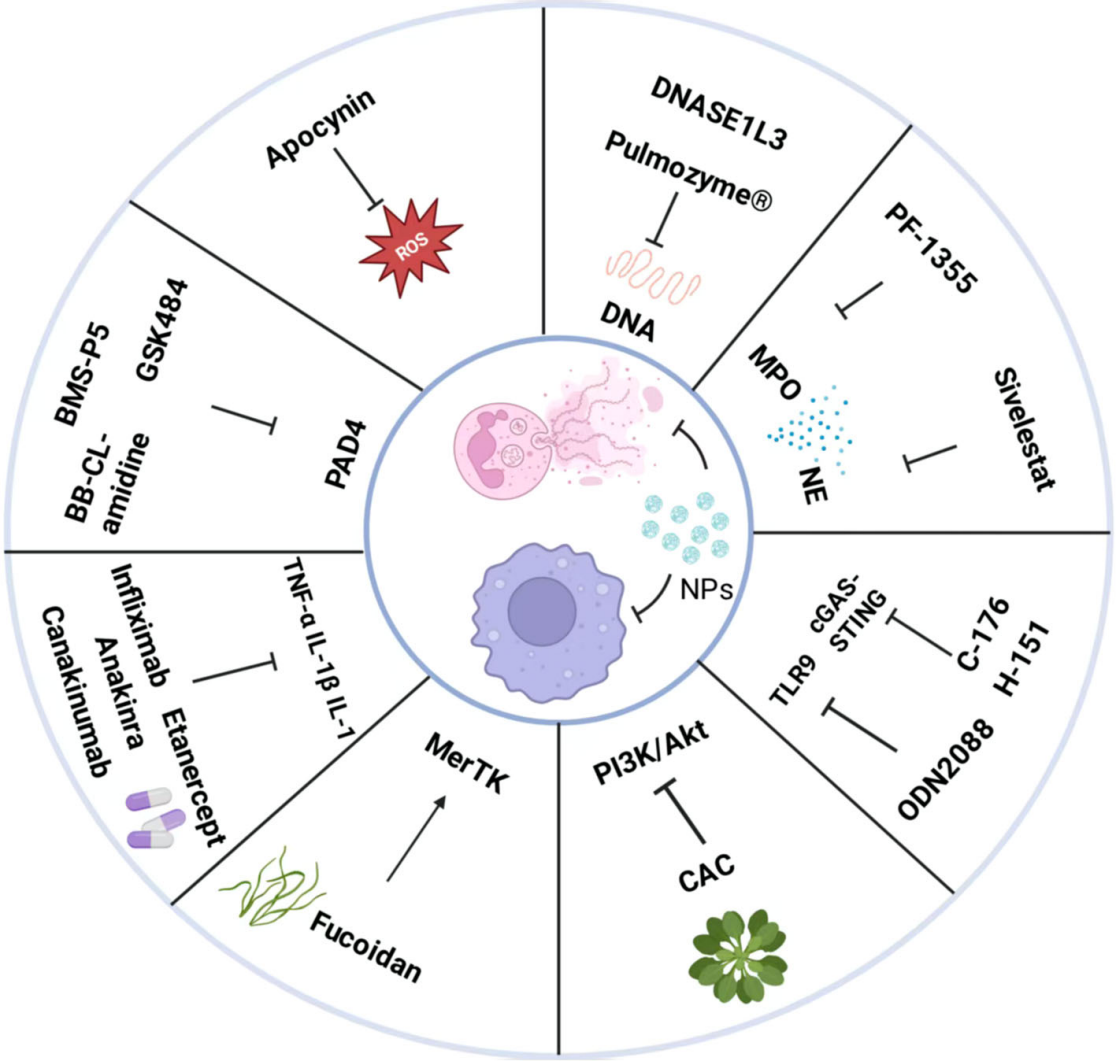
Potential therapeutic targets for NET-macrophage interactions. The left side of the diagram illustrates therapies targeting neutrophil extracellular traps (NETs), including PAD4 inhibitors (GSK484, BB−Cl−Amidine, BMS−P5), NOX/ROS inhibitors (Apocynin), and MPO/NE inhibitors (PF−1355, Sivelestat). NETs clearance strategies involve DNase I (Pulmozyme), DNASE1L3, and nanoparticles (NPs) for targeted delivery to the lesion site. The right side depicts therapies aimed at blocking pro-inflammatory macrophage polarization while enhancing their clearance and repair functions. Macrophage regulation approaches include TLR9 antagonists (ODN−2088), cGAS–STING inhibitors (C−176, H−151), cytokine blockade (targeting TNF−α, IL−1β, IL−6 with agents such as Infliximab, Etanercept, Anakinra, Canakinumab), MerTK activation (Fucoidan), and PI3K/Akt pathway modulation (CAC). Furthermore, multi-target combination strategies are presented: NPs can be utilized for the co-delivery of NETs modulators and macrophage reprogramming agents, or these approaches can be combined with immunotherapies or metabolic therapies.

Potential therapeutic targets for NET-macrophage interactions primarily focus on inhibiting NETosis, disrupting macrophage function, and combination therapies (**Figure 6**).

### 5.1 Targeting NETs

Therapeutic strategies for NETs focus on inhibiting NET formation or promoting their clearance, thereby reducing the abnormal activation of macrophages by NETs at the source. Targeting NETs can be approached from two aspects: inhibiting key molecules involved in NET formation and degrading already formed NETs.

NET formation can be curtailed by targeting key mediators of NETosis. PAD4 inhibitors suppress NETosis upstream by blocking the critical step of histone citrullination (138). Several agents—such as GSK484, BB−Cl−Amidine, and their derivatives—have shown promising efficacy in preclinical models of sepsis, RA, neuroinflammation, and acute respiratory distress syndrome (ARDS) (139–142). A few have progressed to early clinical trials. BMS−P5, a recently developed PAD4 inhibitor, combines enhanced potency, isoform selectivity, and oral bioavailability with minimal off−target activity, positioning it as a leading candidate for clinical evaluation (143). Nevertheless, broad clinical adoption remains challenging due to persistent issues with isoform−specific targeting and immune perturbation. In addition, NOX inhibitors, such as apocynin, can attenuate classical suicidal NETosis by limiting the burst production of ROS (65, 144). However, it should be noted that ROS play a broad role in systemic immune defense, and comprehensive inhibition may increase the risk of infection. Therefore, this target may be more suitable for local or short-term applications. Furthermore, NET−associated histones are highly cytotoxic and can robustly activate macrophages. Neutralizing histones with heparin or anti−histone antibodies has shown protective effects in preclinical models of sepsis and thrombosis (74, 145). MPO and NE—key antimicrobial enzymes embedded in NETs that also drive tissue injury—represent additional druggable nodes; their selective inhibitors can dampen NETosis and downstream damage (146, 147). Most MPO and NE inhibitors remain in preclinical development, but there is supportive evidence that MPO inhibition improves cardiovascular outcomes, and the NE inhibitor sivelestat confers protection in specific disease models. Notably, dual inhibition of MPO and NE may offer additive or synergistic control of neutrophil−mediated inflammation (148–150).

Secondly, degrading preformed NETs is the most direct strategy to reduce their impact on macrophages. As an endonuclease that efficiently degrades extracellular chromatin, DNase I shows broad therapeutic promise. Its primary action is to cleave the DNA scaffold of NETs, thereby reducing NET burden. In experimental models, DNase I attenuates inflammation in rheumatoid arthritis and colitis, though its efficacy in SLE is diminished by NET−stabilizing proteins. Beyond inflammation, DNase I disrupts tumor cell adhesion to vascular endothelium, inhibiting metastatic spread (25, 151, 152). Clinically, recombinant human DNase I (e.g. Pulmozyme^®^) was first approved for cystic fibrosis, where it hydrolyzes airway DNA to improve mucus clearance (153). More recently, it has been explored in trials for cancer−associated thrombosis, metastasis prevention, and inflammatory diseases. Looking ahead, lesion−targeted delivery using nanocarriers or antibody conjugates may enhance efficacy while limiting off−target effects. In addition, certain targeted agents can mediate macrophage clearance of NETs. For example, DNASE1L3, a potential autoantigen, degrades chromatin released from apoptotic cell-derived particles. Studies have shown that DNASE1L3 can interact with macrophages, enhancing phagocytosis to mediate NET degradation and promote NET clearance (154).

### 5.2 Targeting macrophages

This strategy does not directly target NETs themselves, but rather interferes with the recognition of NETs signals by macrophages and their subsequent responses, thereby “disrupting” the circuit. This includes blocking the receptors through which macrophages recognize NETs and interfering with macrophage polarization by modulating cytokine signaling pathways.

One approach is to block the receptors through which macrophages recognize NETs. Macrophages detect NET−derived DNA via TLR9, triggering proinflammatory cytokines such as IL−1β. CpG oligodeoxynucleotide–based TLR9 antagonists (e.g., ODN−2088) have been evaluated in early trials for autoimmune diseases and atherosclerosis to blunt NET−DNA–driven inflammation. Because TLRs are pleiotropic and TLR9 contributes to antiviral defense, therapeutic benefit must be balanced against the risk of immunosuppression (89, 155). The cGAS–STING pathway is another tractable target. NET−released DNA can activate cytosolic cGAS, engage STING, and drive IFN and inflammatory programs. Small−molecule inhibitors of cGAS or STING (H−151, C−176) have shown efficacy in preclinical SLE and lung−injury models by reducing IFN output and downstream inflammation, and several STING inhibitors have entered clinical trials for cancer and autoimmunity. Future opportunities include developing highly selective agents for NET−driven contexts and combining them with NET−formation inhibitors (156, 157). Mer tyrosine kinase (MerTK), a member of the Tyro3-Axl-MerTK receptor tyrosine kinase family, is primarily expressed on phagocytes such as macrophages and is essential for efferocytosis and for inducing anti-inflammatory, reparative phenotypes. Fucoidan (FUC), a sulfated polysaccharide from brown algae with anti-inflammatory, antioxidant, immunomodulatory, and neuroprotective activities, activates the Gas6/MerTK axis in oxaliplatin-treated bone marrow–derived macrophages (BMDMs), a model of chemotherapy-induced peripheral neuropathy (CIPN). This activation upregulates SOCS3 and suppresses neuroinflammation. In co-cultures with LPS-induced NETs, fucoidan further enhances macrophage phagocytosis and promotes NET clearance, thereby mitigating CIPN (158). Additionally, spatiotemporally targeted gene-editing platforms—such as CRISPR–Cas9 delivered via receptor-targeted liposomes or lipoprotein carriers—enable selective delivery to macrophages by exploiting surface receptor recognition. These systems can efficiently edit pathogenic programs within macrophages; in the tumor microenvironment, for example, they can reprogram “educated” pro-tumor macrophages, restore antitumor activity, and disrupt immune evasion (159, 160).

Modulating cytokine signaling to alter macrophage polarization can directly ameliorate inflammation or reduce NET formation. Biologics that target proinflammatory cytokines—TNF−α inhibitors, IL−6 receptor antagonists, and IL−1β inhibitors—are used in inflammatory conditions such as MI and RA (161, 162). By suppressing M1−like polarization and downstream inflammatory cascades, they indirectly disrupt the NET–macrophage circuit. In clinical studies, canakinumab (anti−IL−1β) and anakinra (IL−1 receptor antagonist) have been evaluated in MI, where they dampened macrophage−associated inflammation and reduced post−MI cardiovascular events (163, 164). In contrast, results with anti−TNF−α agents (infliximab, etanercept) in heart failure have been inconsistent, with some trials suggesting potential worsening of disease—underscoring the need for precise timing and patient selection (165). These cytokine−targeted strategies are also well supported preclinically. In a rat arthritis model, an IL−6R/TNF−α bispecific antibody (fenobody) neutralized TNF−α cytotoxicity by inhibiting phosphorylation of IκBα and p65 in the NF−κB pathway and JAK−dependent phosphorylation of STAT3 (162). In a monocrotaline−induced pulmonary arterial hypertension (PAH) rat model, an IL−6R antagonist improved hemodynamics and reduced tissue remodeling (166). Despite broad evidence of efficacy, challenges remain, including interindividual variability, secondary loss of response, and infection risk. Future priorities include identifying predictive biomarkers of benefit and developing inhibitors that target NET−associated inflammatory pathways with greater precision. In addition, Cayratia albifolia CLLi (CAC), is a traditional Chinese medicinal herb, known as “jiao’mei’gu” in China. Studies have found that CAC extract inhibits macrophage-mediated inflammatory responses and reduces NETosis through the PI3K/Akt pathway, significantly alleviating rheumatoid arthritis (167).

### 5.3 Multi-target combination therapies

Given the complexity and redundancy of the NETs-macrophage circuit, combination therapy is likely the most effective and durable strategy. In cancer therapy, targeting NETs Combined with Macrophage Reprogramming Enhances Therapeutic Efficacy. For instance, in cervical cancer with lymph node metastasis, targeted inhibition of NETs by DNase I combined with modulation of the CXCL9/CXCR3 axis can enhance the efficacy of PD-1/PD-L1 inhibitors and restore anti-tumor immunity (168, 169). In metabolic diseases, metformin combined with NETs-modulating agents can exert synergistic effects by improving macrophage function (170). Furthermore, nanoparticles (NPs) can be used for targeted delivery of NETs modulators (e.g., NE inhibitors) or macrophage polarization regulators (e.g., PI3K/AKT pathway inhibitors), enabling precise modulation of their interaction (171, 172).

Currently, although some progress has been made in developing drugs targeting the interaction between NETs and macrophages, numerous challenges remain, such as lack of specificity and suboptimal long-term efficacy, which require further practice and investigation by clinicians and researchers.

## 6 Conclusion

Inflammation, as a common pathophysiological basis for various diseases, hinges on a sophisticated and intricate regulatory network among immune cells. Neutrophils and macrophages, as key effector cells of the innate immune system, engage in a dynamic interactive network mediated by NETs. This network profoundly influences the progression of inflammatory responses and plays a pivotal role in the pathogenesis of major diseases, including autoimmune diseases, cardiovascular diseases, and cancer. Current research indicates that NETs extend beyond their traditional antimicrobial defense functions, establishing a highly context-dependent regulatory mode with macrophages. Under physiological conditions, this mode maintains the balance between immune homeostasis and tissue healing; whereas, in pathological states, it evolves into a significant mechanism driving tissue damage and disease progression. Current studies have preliminarily elucidated the core molecular mechanisms of their interaction. NETs, through their unique molecular composition—including citrullinated histones, mitochondrial DNA, and granule proteins—act as crucial signaling vehicles that regulate macrophage polarization and functional properties (173). Conversely, macrophages modulate the formation and clearance of NETs through pathways such as cytokine secretion, regulation of oxidative stress levels, and phagocytosis (174). This bidirectional regulation manifests distinct pathological features in different disease contexts. In autoimmune diseases, it presents as a vicious cycle of autoantigen exposure and impaired clearance; in cardiovascular diseases like atherosclerosis, it accelerates plaque instability and impedes myocardial repair; within the tumor microenvironment, it often promotes tumor metastasis and immune evasion.

However, this research field still faces several significant challenges. Key questions remain unresolved: Under what microenvironmental signals do NETs drive macrophages toward an M1 or M2 phenotype? What are the underlying mechanisms of metabolic reprogramming and epigenetic regulation? Furthermore, the criteria for distinguishing “physiological” from “pathological” NETs, as well as the mechanisms by which macrophages differentially recognize them, remain poorly understood and represent significant gaps in current knowledge (54, 175). Furthermore, in chronic inflammatory settings or tumor microenvironments, the NET–macrophage axis likely evolves dynamically over time. How these dynamics shape disease progression and therapeutic responsiveness remains largely undefined, due in part to the scarcity of long−term disease models and clinical longitudinal studies (176). Most mechanistic insights come from ex vivo co−cultures or animal models, which only partially recapitulate the human immune milieu and the chronic course of disease. Although single−cell and spatial transcriptomic approaches have revealed substantial cellular heterogeneity, technologies for real−time, high−resolution, *in vivo* visualization of NET–macrophage interactions are still lacking, limiting mechanistic precision and translational relevance. Intercellular crosstalk also comprises concurrent and sequential events among multiple lineages, which we did not systematically address here. For example, B cells in pericardial adipose tissue contribute to the emergency recruitment of neutrophils after myocardial infarction (177). Future work will integrate longitudinal human datasets, advanced imaging, and multi−omic perturbation to resolve these dynamics and address these gaps.

Correspondingly, methodological innovation is key to overcoming these bottlenecks. The application of single-cell multi-omics technologies, such as single-cell RNA sequencing combined with ATAC-seq, enables the simultaneous analysis of macrophage transcriptional states, chromatin accessibility, and their responses to NET components at single-cell resolution. Spatial transcriptomics can untangle the spatial adjacency and molecular crosstalk between NETs and macrophages within specific regions of tissue pathology *in situ*. Furthermore, the development of genetic tools capable of specifically labeling, modulating, or depleting NETs or specific macrophage subsets *in vivo* (e.g., gene knock-out or overexpression mouse models) will allow for the direct validation of their causal roles and enable dynamic intervention.

A comprehensive understanding of NETs-macrophage interactions provides guidance for the development of next-generation immunotherapies. Therapeutic strategies should shift from broad-spectrum “blockade” to precise “modulation.” While directly targeting NETs (e.g., using PAD4 inhibitors or DNase I) is effective, it may compromise host defense mechanisms. Future directions lie in developing more specific interventions, such as: engineering enzymes capable of selectively degrading pathological versus normal physiological NETs; or developing nanodrug delivery systems that target diseased sites to deliver reprogramming signals to macrophages, thereby shifting their phenotype from pro-inflammatory/pro-tumorigenic to anti-inflammatory/anti-tumorigenic. Furthermore, combination therapies hold significant promise. For instance, combining NETs inhibition with immune checkpoint blockade, macrophage checkpoint inhibitors (e.g., anti-CD47 antibodies), or cell therapies has demonstrated synergistic effects in preclinical models. The core principle is to achieve precise temporal, spatial, and cell-specific control of treatment based on an accurate interpretation of the molecular language governing these interactions.

In-depth investigation of the interaction between NETs and macrophages will not only help untangle the shared pathophysiology of numerous diseases but also provide novel insights for re-establishing immune homeostasis. Future therapeutic advances will largely depend on our ability to leverage tools such as single−cell and spatial multi−omics, nanotechnology, and gene editing to precisely decipher the crosstalk between NETs and macrophages, and ultimately to develop dynamic, tunable, and precise intervention strategies targeting specific disease stages and specific cellular subsets. This not only represents the main research direction in this field, but also serves as a critical bridge connecting fundamental discoveries with clinical translation.
