# Correction: Interactions between neutrophil extracellular traps and macrophages: the key to inflammatory diseases

**DOI:** 10.3389/fimmu.2026.1859054

**Published:** 2026-04-24

**Authors:** Xiaoyu Shan, Xiaodong Fan, Xiaofei Geng, Yongchun Liang, Yingxi Yang, Junping Zhang

**Affiliations:** 1 First Teaching Hospital of Tianjin University of Traditional Chinese Medicine, Tianjin, China; 2 National Clinical Research Center for Chinese Medicine, Tianjin, China; 3 College of Traditional Chinese Medicine, Tianjin University of Traditional Chinese Medicine, Tianjin, China; 4 Information and Technology School, Tianjin College of Commerce, Tianjin, China; 5 Tianjin Key Laboratory of Modern Chinese Medicine Theory of Innovation and Application, Tianjin, China

**Keywords:** cellular communication, immune inflammation, innate immunity, macrophages, neutrophil extracellular traps

Affiliation “College of Traditional Chinese Medicine, Tianjin University of Traditional Chinese Medicine, Tianjin, China”, was omitted for author “Xiaoyu Shan”. This affiliation has now been added for author “Xiaoyu Shan”.

Affiliation “^2^National Clinical Research Center for Chinese Medicine, Tianjin, China”, was omitted for the authors “Xiaofei Geng, Yongchun Liang, Junping Zhang”. This affiliation has now been added for author “Xiaofei Geng, Yongchun Liang, Junping Zhang”.

The order of authors in the author list of the published paper was erroneously given as:

“Xiaoyu Shan, Xiaodong Fan, Xiaofei Geng, Yongchun Liang, Junping Zhang and Yingxi Yang”

The correct author list reads:

“Xiaoyu Shan, Xiaodong Fan, Xiaofei Geng, Yongchun Liang, Yingxi Yang and Junping Zhang”

The **Author contributions** statement was erroneously given as “XS: Writing – original draft. XF: Supervision, Visualization, Writing – review & editing. XG: Supervision, Visualization, Writing – review & editing. YL: Supervision, Visualization, Writing – review & editing. JZ: Supervision, Visualization, Writing – review & editing. YY: Funding acquisition, Supervision, Visualization, Writing – review & editing”.

The correct **Author contributions** statement is “XS: Writing – original draft. XF: Supervision, Visualization, Writing – review & editing. XG: Supervision, Visualization, Writing – review & editing. YL: Supervision, Visualization, Writing – review & editing. YY: Funding acquisition, Supervision, Visualization, Writing – review & editing. JZ: Funding acquisition, Supervision, Visualization, Writing – review & editing”.

And finally, Author “Junping Zhang” was erroneously omitted as corresponding author. The correct corresponding authors are “Yingxi Yang and Junping Zhang”.

The original version of this article has been updated.

